# Editorial: Kidney and heart cross-talk

**DOI:** 10.3389/fneph.2025.1598135

**Published:** 2025-04-11

**Authors:** Nicoletta Mancianti, Marta Calatroni, Giacomo Deferrari, Edoardo La Porta

**Affiliations:** ^1^ Department of Medical Science, Nephrology, Dialysis and Transplantation Unit, University Hospital of Siena, Siena, Italy; ^2^ Department of Biomedical Sciences, Humanitas University, Milan, Italy; ^3^ Department of Cardionephrology, Istituto Clinico Ligure di Alta Specialità (ICLAS), GVM Care and Research, Rapallo, Italy; ^4^ UO Nephrology Dialysis and Transplant, IRCCS Istituto Giannina Gaslini, Genoa, Italy; ^5^ UOC Nephrology, Dialysis and Trasplantation, UOSD Dialysis IRCCS Istituto Giannina Gaslini, Genoa, Italy

**Keywords:** cardiorenal syndrome, chronic kidney disease progression, hemodialysis and cardiovascular risk, kidney transplantation, biomarkers in nephrology

Two organs, one fate: the intricate interplay between the heart and kidneys is not a simple equation, but a complex, bidirectional symphony in which dysfunction in one precipitates deterioration in the other. This reality highlights the strong connection between chronic kidney disease (CKD) and cardiovascular disease (CVD). Patients with CKD face a cardiovascular risk that is significantly higher than that of the general population. CKD and CVD share common underlying mechanisms, including chronic inflammation, endothelial dysfunction, oxidative stress, metabolic dysregulation, and changes in gut microbiota. These factors contribute to the markedly increased cardiovascular risk in patients with CKD. However, management of this risk is often fragmented, indicating the need for a multidisciplinary and integrated approach.

Our Research Topic has compiled key studies that explore these shared mechanisms, offering new perspectives on emerging biomarkers, therapeutic strategies, and physiological interactions. For instance, Kaysi et al. demonstrated how pulmonary and systemic congestion in hemodialysis patients impacts cardiovascular mortality, emphasizing the importance of proactive volume management (1). Similarly, Rajnochova Bloudickova et al. highlighted the need for more effective risk stratification in kidney transplant recipients, while Zhao et al. assessed the diagnostic value of high-sensitivity cardiac troponin T in dialysis patients with myocardial infarction, underscoring the role of biomarkers in early diagnosis (2).

Systemic inflammation is another critical factor in the simultaneous involvement of the heart and kidneys. Orsi et al. analyzed multisystem inflammatory syndrome in children with associated proximal tubular injury, demonstrating how inflammation can have multi-organ effects (3). Meanwhile, Kim et al. showed that uncontrolled hypertension in kidney transplant recipients increases the risk of graft failure, reinforcing the importance of strict blood pressure monitoring. The link between hypertension and renal dysfunction is well-documented, as elevated blood pressure contributes to glomerular injury, worsening kidney function, and subsequently increasing cardiovascular morbidity (4).

Early diagnosis of cardiovascular complications in patients with CKD is another crucial aspect. Su et al. developed a risk model for the early detection of acute myocardial infarction in nephropathic patients, which improved the predictive ability over traditional models. Kwon et al. demonstrated that components of the metabolic syndrome influence the risk of adverse renal outcomes in patients with atrial fibrillation, suggesting an integrated approach to the management of comorbidities. Wu et al. investigated the relationship between early-stage renal insufficiency and cardiac structural and functional abnormalities in a large population of asymptomatic Asians, indicating that even minor renal alterations may have significant cardiac implications. Finally, Molina Andújar et al. examined the impact of cardiac surgery-associated acute kidney injury on one-year major adverse kidney events, emphasizing the need for more effective preventive strategies in patients undergoing cardiovascular interventions (5).

These studies underscore the crucial role of various pathophysiological factors in the cardiorenal syndrome. Chronic inflammation and oxidative stress accelerate endothelial damage and disease progression, while mitochondrial dysfunction compromises energy metabolism, exacerbating both heart and kidney failure. Endothelial dysfunction and vascular calcification increase the risk of major cardiovascular events, while gut microbiota alterations contribute to systemic inflammation and organ damage. Managing pulmonary and systemic congestion in hemodialysis patients is essential to reducing mortality and improving quality of life.

A growing body of research suggests that mitochondrial dysfunction plays a pivotal role in cardiorenal syndrome. The inability of mitochondria to maintain cellular energy production leads to increased oxidative stress, which further exacerbates endothelial damage and metabolic disturbances. These dysfunctions set off a cascade of events that contribute to both renal and cardiac deterioration. Additionally, alterations in calcium-phosphate metabolism have been linked to vascular calcification, a major risk factor for cardiovascular mortality in patients with CKD.

Despite advancements in research, managing cardiovascular risk in patients with CKD remains challenging. The lack of an effective multidisciplinary approach and the underutilization of advanced biomarkers limit the efficacy of preventive strategies. Moreover, risk stratification often occurs only after cardiovascular symptoms appear, diminishing the impact of early intervention. Personalized therapies remain an ongoing challenge, as many cardiovascular treatments fail to account for the specificities of CKD.

To improve clinical outcomes, an innovative approach is required that includes closer collaboration among specialists, extensive use of biomarkers to refine diagnosis, and new therapeutic strategies such as SGLT2 inhibitors and finerenone to mitigate cardiovascular risk in nephropathic patients. Optimizing dialysis and transplantation management, incorporating cardiovascular risk considerations, is equally essential to enhance prognosis. Furthermore, therapeutic approaches targeting chronic inflammation and oxidative stress should be integrated into standard treatment protocols to address the underlying molecular pathways contributing to cardiorenal syndrome.

Artificial intelligence and machine learning are also emerging as powerful tools for risk stratification and early diagnosis in patients with CKD. Predictive models utilizing large datasets can help identify high-risk individuals and personalize treatment plans, leading to improved outcomes. The integration of these technologies into clinical practice could revolutionize the way cardiovascular and renal risks are managed in the future.

These emerging insights present exciting new interventional possibilities and underscore the urgency of rethinking our cardiovascular approach to patients with CKD. Nephrology and cardiology can no longer exist in isolation. The future of cardiovascular medicine for CKD patients lies in a unified, integrative approach. Addressing CKD without taking the heart into account is like navigating a storm without a compass—eventually, the course will be lost.
